# Non-human primates and *Leishmania* immunity

**DOI:** 10.1016/j.cytox.2020.100038

**Published:** 2020-10-12

**Authors:** Sonia André, Vasco Rodrigues, Morgane Picard, Ricardo Silvestre, Jérôme Estaquier

**Affiliations:** a INSERM-U1124, Paris University, Paris, France; b ICVS/3B’s – PT Government Associate Laboratory, Braga/Guimarães, Portugal; c Life and Health Sciences Research Institute (ICVS), School of Medicine, University of Minho, Braga, Portugal; d Centre de Recherche du CHU de Québec, Laval University, QC, Quebec, Canada

**Keywords:** Leishmania, Monkeys, Immunity, T cells, Tfh, CXCR5, Cytokines

## Abstract

In the context of infectious diseases, non-human primates (NHP) provide the best animal models of human diseases due to the close phylogenetic relationship and the similar physiology and anatomical systems. Herein, we summarized the contribution of NHP models for understanding the immunity to leishmaniases, which are a group of diseases caused by infection with protozoan parasites of the genus *Leishmania* and classified as one of the neglected tropical diseases.

## 1 Introduction

The leishmaniases are a group of diseases caused by infection with protozoan parasites of the genus *Leishmania,* affecting more than 98 countries, and are classified as one of the neglected tropical diseases (NTD). Worldwide, the population of 350 million is at risk with an annual incidence of 0.7–1.2 million cases of cutaneous leishmaniasis (CL) and 0.2–0.4 million cases of visceral leishmaniasis (VL). VL is the most severe form of leishmaniasis and is caused by *Leishmania infantum* or *L. donovani* leading to the infection of spleen, liver and bone marrow*.* The disease is transmitted to various mammals through the bites of infected sandflies. Thus, the domestic dog is the main urban reservoir for VL and they have been used as model for the study of VL disease [1], [2], [3]. However, the immunological tools to study canine VL are limited. Inbred mice strains have been useful to investigate the mechanisms of adaptive and innate immune responses but invariably control infections with viscerotropic *Leishmania* species and develop a life-long latent infection [4]. Moreover, mice and humans differ in number of effector molecules such as defensin [5], [6] and granulysin [7] and the capacity of human phagocytic cells to produce NO appears to be under much tighter regulation [8], [9]. Furthermore, no infection of intestine is observed in mouse model [10].

Therefore, despite the usefulness of these models, new insights into the immunopathogenesis of VL would benefit from a more frequent employment of alternative animal models such as non-human primates (NHP) that constitute powerful experimental models for understanding host-pathogen interactions in humans.

1) Non-human primates and human infectious diseases

**Fig. 1:**
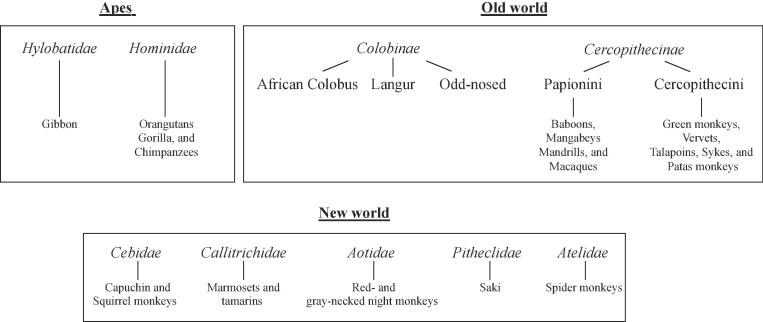
Primate phylogeny.

The phylogenetic closeness of non-human primates (NHP) to humans makes them attractive models for assessing the pathogenicity related to infectious diseases, as well as for the development of vaccine and drug therapies. New World primate species include the families of *Cebidae*, *Callitrichidae*, *Aotidae*, *Pitheclidae*, and *Atelidae* (Fig. 1). Apes (*Hominoidea* and *Hylobatidae*) and Old World monkeys are native to either Africa or Asia. The latter are subdivided into two distinct subfamilies: *Colobinae* and *Cercopithecinae* [11]. Of interest, experimental microbe infections have demonstrated distinct susceptibility of non-human primate species suggesting host-factors associated [12], [13], [14]. In the context of infectious diseases, the use of great apes has been discontinued, mostly due to ethical reasons. Macaques (member of the *Cercopithecinae* family*)* have been extensively explored over the past century as models of infections caused by bacteria, virus, parasites or prions. A major boost in the use of macaques for studies on the immunology of infection was driven by the emergence of the AIDS pandemic in the mid-80′s. Soon after, it was discovered that macaques infected with the HIV closely-related simian immunodeficiency virus (SIV) developed a progressive immunodeficiency similar to AIDS [15], [16].

2) Non-human primate models and leishmania

a) Visceral leishmaniasis: In 1924, Shortt *et al.* reported an experimental infection of Old World primates by intradermal inoculation of splenic puncture material from an Indian patient with Kala-azar [17], [18]. This was reproduced two years later by Greig *et al.* [19]. Experimental infections of vervets also cause visceral organ enlargement and parasitosis following intraperitoneal, intranasal or intradermal *Leishmania* inoculation [20], [21]. Experiments performed thereafter clearly demonstrated that various monkeys living in Africa including sykes and baboons can be infected experimentally with *L. donovani* or *L. infantum*, showing low grade infections for periods ranging between four and eight months followed by evidence of spontaneous cure [22], [23], [24]. Furthermore, infection of (Presbytis entellus) a langur monkey was demonstrated to be a model for *L. donovani* [25], [26], [27]. In contrary, experiments performed in New World monkeys including Aotus monkeys [28], [29], squirrel monkeys [30] and marmosets [31], demonstrated fulminating VL after intravenous or intraperitoneal inoculation of amastigotes. Due to the rapid disease development, New World species have been useful for drug testing [32], [33]. Finally, disease mimicking human VL was established in macaques. These animals developed a systemic disease showing characteristic features of human VL such as fever, diarrhea, body weight loss, anemia, hypergammaglobulinemia and transient lymphocytosis, as well as lymph node, liver and spleen enlargement [34]. Thus, although macaques were less susceptible to develop fulminant infections as compared to new world species, they are considered a model for pre-clinical evaluation of novel chemotherapeutics or vaccine candidates for human VL.

b) Cutaneous leishmaniasis: Vervet monkeys can be also animal model for studying *L. major* infection [35], [36], [37], [38]. Like infected individuals with leishmaniasis, vervets have been shown to undergo spontaneous cure following experimental infection with *L. major*. Furthermore, *L. braziliensis* infection of macaques resulted in localized skin ulcerations, but complete spontaneous clinical healing occurred in infected animals. The lesion development was variable depending on the challenging parasite [39], [40], [41], [42], [43], dose and route of exposure [39], [44]. Furthermore, monkeys infected with *L. major* transmitted by *Phlebotomus papatasi* [45] developed skin lesions longer than infections induced by needle inoculation [40]. Hence, monkeys have been considered to be useful for studying the interactions between parasite and host determinants for leishmania infection as well as for the evaluation of new drugs and candidate vaccines for human disease [46].

3) Immune responses in *Leishmania*-infected NHP

Primate models have been used to study host responses to *leishmania* [47], [48], [49], [50], [51], [52], [53]. Of interest, studies with the vervet model for cutaneous leishmaniasis have demonstrated that resistance is correlated with increased production of gamma interferon (IFN-γ) and strong delayed type hypersensitivity (DTH) responses similar to those seen in human patients with cutaneous leishmaniasis [54], [55]. Macaques infected with *L. major* developed a typical T helper (Th) cytokine profile related to type 1-mediated immunity (Th1 cells expressing IFN-γ) in which IL-12 was observed to improve immunity [40], [56], [57], [58]. It has been also shown that experimental infection of macaques with *L. braziliensis* induced the recruitment and activation of inflammatory mast cells, granulocytes, mononuclear phagocytes, and lymphocytes at the site of infection. Longitudinal characterization of immune response in macaques revealed in the chronic phase, persisting parasites induced a Th1 profile associated with granulomatous reaction. In addition, less differentiated macrophages are observed, forming mature tissue granulomas, which are then substituted by fibroblasts resulting in fibrosis [43]. After 8 weeks, CD8 T cell numbers increased in healing lesions and expressed both TNF-α and IFN-γ, contributing to the clearance of parasites. Furthermore, IL-10-producing CD4^+^CD25^+^ T cells also accumulate in self-healing skin lesions [43], [59] and promote lesional granuloma maintenance [60]. Whereas the immune parameters associated with immune protection in NHP with cutaneous leishmaniasis (CL) remains elusive [57], [61], it has been shown that macaques immunized with the sand-fly PdSP15 salivary protein are protected against cutaneous leishmaniasis [62]. The administration of pentavalent antimonial sodium stibogluconate (20 mg/kg for 20 days) in infected macaques, reduced the lesion severity and accelerated healing, which was associated with the modulation in the expression of hundred genes in the skin biopsies from the lesion site [63]. These findings are in agreement with data from human biopsies, which demonstrated that treatment failure was linked to the excessive activation of the cytolytic pathway activated during infection [64], [65]. Additional studies in macaques could also address the role of tryptophan-2,3-deoxygenase (TDO), that has recently been described to inhibit parasite burden in human lesions and cultured macrophages [65].

Whereas most of these monkey studies are informative about CL, few of them have addressed immune dysregulation following visceral leishmaniasis. It has been shown that vaccine responses reduce inflammation and structural changes of the splenic white pulp caused by *L. donovani* infection in macaques [66]. Recently we provided major advances regarding immune dysregulation associated with *L. infantum* in macaques [67]. Thus, the infection was associated with the differentiation of splenic CD4 T cells, which became more sensitive to FasL-mediated cell death [68], [69], [70]. Early after infection, CD4 T cells were Th1 polarized, switching thereafter to an IL-10 dominated profile consistent with observations in humans and rodents [71], [72], [73], [74], [75], [76], [77]. Most importantly, associated with the disruption of splenic architecture, we found that a population that expressed CXCR5 and PD-1, named T follicular helper cells (Tfh) [67], [78], [79], [80], [81], [82], were abnormally differentiated and not sustained during the chronic phase. The expression of the master transcriptional factor Bcl-6 of Tfh and the cytokine IL-21 that is critical for B cell activation [83], [84], were lowered during the chronic phase. This is of crucial interest given that concomitantly with abortive Tfh differentiation, B cells failed to mature and the circulating levels of parasite-specific IgG and IgM were low, despite the chronic persistence of hypergammaglobulinemia [67], [70].

In addition, we observed in macaque infected with *L. infantum* an expansion of splenic CD8 T cells. During experimental VL in mice, the splenic CD8 T cell population expands several-fold at two months post-infection [85], [86], [87]. Whereas the role of IFN-γ in promoting the killing of intracellular amastigotes is well documented, the role of effector cytotoxic molecules remains less clear, particularly during VL. Thus, the cytotoxic molecule granulysin that kills intracellular parasites in association with granzyme and perforin is absent in mice [7], which further calls for the need of more accurate models of human VL, such as NHP where granulysin is expressed [88]. Thus, NHP may constitute powerful experimental models for understanding host-pathogen interactions that are not directly accessible in human patients, particularly concerning deep tissues in which immune dysregulation may favor parasite persistence as well as the early events after infection, which are usually poorly characterized in infected individuals. They also offer the opportunity to evaluate immunotherapies based on interleukins or antibodies directed against molecules that contribute to the regulation of immune system. Thus, the development of the tools for monkeys in the context of HIV-infection may represent an opportunity for assessing novel strategies for VL/CL diseases.

## 2 Conclusions

Anti-leishmanial drugs have been demonstrated to control parasite infection. However, the observation that the relapse rate is increasing in Asia [89], [90] and in Africa, particularly in patients co-infected with HIV [91], [92], [93] indicates the requirement for an effective immunity in the control of leishmania. Parasite breakthrough increases the potential for the emergence of resistant parasites [94], [95], [96]. Therefore, monkeys represent attractive models for assessing pathogenicity, processes associated with parasite relapse and novel therapies for leishmaniasis, which is one of the main tropical neglected diseases.

## Declaration of Competing Interest

The authors declare that they have no known competing financial interests or personal relationships that could have appeared to influence the work reported in this paper.

## References

[1] da Silva A.V.A., Figueiredo F.B. (2018). Morphophysiological changes in the splenic extracellular matrix of Leishmania infantum-naturally infected dogs is associated with alterations in lymphoid niches and the CD4+ T cell frequency in spleens. PLoS Negl Trop Dis.

[2] Falqueto A., Ferreira A.L. (2009). Cross-sectional and longitudinal epidemiologic surveys of human and canine Leishmania infantum visceral infections in an endemic rural area of southeast Brazil (Pancas, Espirito Santo). Am J Trop Med Hyg.

[3] Petersen C.A. (2009). Leishmaniasis, an emerging disease found in companion animals in the United States. Top Companion Anim Med.

[4] Nieto A., Domínguez-Bernal G. (2011). Mechanisms of resistance and susceptibility to experimental visceral leishmaniosis: BALB/c mouse versus Syrian hamster model. Vet Res.

[5] Risso A. (2000). Leukocyte antimicrobial peptides: multifunctional effector molecules of innate immunity. J Leukoc Biol.

[6] Mestas J., Hughes C.C. (2004). Of mice and not men: differences between mouse and human immunology. J Immunol.

[7] Dotiwala F., Mulik S. (2016). Killer lymphocytes use granulysin, perforin and granzymes to kill intracellular parasites. Nat Med.

[8] Murray H.W., Cartelli D.M. (1983). Killing of intracellular Leishmania donovani by human mononuclear phagocytes. Evidence for oxygen-dependent and -independent leishmanicidal activity. J Clin Invest.

[9] Kröncke K.D., Fehsel K. (1998). Inducible nitric oxide synthase in human diseases. Clin Exp Immunol.

[10] Lewis M.D., Paun A. (2020). Fatal progression of experimental visceral leishmaniasis is associated with intestinal parasitism and secondary infection by commensal bacteria, and is delayed by antibiotic prophylaxis. PLoS Pathog.

[11] Finstermeier K., Zinner D. (2013). A mitogenomic phylogeny of living primates. PLoS One.

[12] Cumont M.C., Monceaux V. (2007). TGF-beta in intestinal lymphoid organs contributes to the death of armed effector CD8 T cells and is associated with the absence of virus containment in rhesus macaques infected with the simian immunodeficiency virus. Cell Death Differ.

[13] Estaquier J., Idziorek T. (1994). Programmed cell death and AIDS: significance of T-cell apoptosis in pathogenic and nonpathogenic primate lentiviral infections. Proc Natl Acad Sci U S A.

[14] Silvestri G., Sodora D.L. (2003). Nonpathogenic SIV infection of sooty mangabeys is characterized by limited bystander immunopathology despite chronic high-level viremia. Immunity.

[15] Letvin N.L., Eaton K.A. (1983). Acquired immunodeficiency syndrome in a colony of macaque monkeys. Proc Natl Acad Sci U S A.

[16] Letvin N.L., Aldrich W.R. (1983). Experimental transmission of macaque AIDS by means of inoculation of macaque lymphoma tissue. Lancet.

[17] Shortt H.E. (1923). The Pathology of Acute Experimental Kala-Azar in Monkeys. Indian J Med Res.

[18] Shortt H.E., Swaminath C.S. (1925). Systemic Infection of a Monkey (Macacus rhesus) by Intradermal Inoculation of Spleen Puncture Material from a Case of Indian Kala-Azar. Indian J Med Res.

[19] Greig E.D.W., Christophers S.R. (1925). Infection of a Monkey (Macacus rhesus) with the Parasite of Indian Kala-Azar following the Introduction of Infective Material into the Lumen of the Small Intestine. Indian J Med Res.

[20] Kirk R. (1942). Studies in Leishmaniasis in the Anglo-Egyptian Sudan. V.-Cutaneous and Mucocutaneous Leishmaniasis. Trans R Soc Trop Med Hyg.

[21] Kirk R. (1945). Studies in Leishmaniasis in the Anglo-Egyptian Sudan. VII. Espundia in a Monkey Infected Experimentally with Sudan Kala-Azar. Trans R Soc Trop Med Hyg.

[22] Githure J.I., Shatry A.M. (1986). The suitability of East African primates as animal models of visceral leishmaniasis. Trans R Soc Trop Med Hyg.

[23] Binhazim A.A., Shin S.S. (1993). Comparative susceptibility of African green monkeys (Cercopithecus aethiops) to experimental infection with Leishmania leishmania donovani and Leishmania leishmania infantum. Lab Anim Sci.

[24] Gicheru M.M., Olobo J.O. (1995). Visceral leishmaniasis in ververt monkeys: immunological responses during asymptomatic infections. Scand J Immunol.

[25] Anuradha, Pal R. (1992). The Indian langur: preliminary report of a new nonhuman primate host for visceral leishmaniasis. Bull World Health Organ.

[26] Misra A., Dube A. (2002). Establishment of asymptomatic Leishmania donovani infection in Indian langurs (Presbytis entellus) through intradermal route. Indian J Exp Biol.

[27] Misra A., Dube A. (2004). Immune responses in normal Indian langur monkeys (Presbytis entellus)–a primate model for visceral leishmaniasis. J Med Primatol.

[28] Chapman W.L., Hanson W.L. (1981). Leishmania donovani in the owl monkey (Aotus trivirgatus). Trans R Soc Trop Med Hyg.

[29] Chapman W.L., Hanson W.L. (1983). Toxicity and efficacy of the antileishmanial drug meglumine antimoniate in the owl monkey (Aotus trivirgatus). J Parasitol.

[30] Chapman W.L., Hanson W.L. (1981). Visceral leishmaniasis in the squirrel monkey (Saimiri sciurea). J Parasitol.

[31] Marsden P.D., Cuba C.C. (1981). Experimental Leishmania chagasi infections in the marmoset Callithrix jacchus jacchus. Trans R Soc Trop Med Hyg.

[32] Madindou T.J., Hanson W.L. (1985). Chemotherapy of visceral leishmaniasis (Leishmania donovani) in the squirrel monkey (Saimiri sciureus). Ann Trop Med Parasitol.

[33] Berman J., Hanson W. (1986). Antileishmanial activity of liposome-encapsulated amphotericin B in hamsters and monkeys. Antimicrob Agents Chemother.

[34] Porrozzi R., Pereira M.S. (2006). Leishmania infantum-induced primary and challenge infections in rhesus monkeys (Macaca mulatta): a primate model for visceral leishmaniasis. Trans R Soc Trop Med Hyg.

[35] Githure J.I., Reid G.D. (1987). Leishmania major: the suitability of East African nonhuman primates as animal models for cutaneous leishmaniasis. Exp Parasitol.

[36] Lawyer P.G., Githure J.I. (1990). Experimental transmission of Leishmania major to vervet monkeys (Cercopithecus aethiops) by bites of Phlebotomus duboscqi (Diptera: Psychodidae). Trans R Soc Trop Med Hyg.

[37] Olobo J.O., Anjili C.O. (1995). Vaccination of vervet monkeys against cutaneous leishmaniosis using recombinant Leishmania 'major surface glycoprotein' (gp63). Vet Parasitol.

[38] Olobo J.O., Reid G.D. (1993). Delayed-type hypersensitivity responses in vervet monkeys self-cured from experimental cutaneous leishmaniasis. Acta Trop.

[39] Amaral V.F., Ransatto V.A. (1996). Leishmania amazonensis: the Asian rhesus macaques (Macaca mulatta) as an experimental model for study of cutaneous leishmaniasis. Exp Parasitol.

[40] Amaral V.F., Teva A. (2001). Leishmania (Leishmania) major-infected rhesus macaques (Macaca mulatta) develop varying levels of resistance against homologous re-infections. Mem Inst Oswaldo Cruz.

[41] Teva A., Porrozzi R. (2003). Leishmania (Viannia) braziliensis-induced chronic granulomatous cutaneous lesions affecting the nasal mucosa in the rhesus monkey (Macaca mulatta) model. Parasitology.

[42] Teva A., Porrozzi R. (2005). Responses of Leishmania (Viannia) braziliensis cutaneous infection to N-methylglucamine antimoniate in the rhesus monkey (Macaca mulatta) model. J Parasitol.

[43] Souza-Lemos C., De-Campos S.N. (2008). Dynamics of immune granuloma formation in a Leishmania braziliensis-induced self-limiting cutaneous infection in the primate Macaca mulatta. J Pathol.

[44] Lujan R., Chapman W.L. (1986). Leishmania braziliensis: development of primary and satellite lesions in the experimentally infected owl monkey. *Aotus trivirgatus.* Exp Parasitol.

[45] Probst R., Wellde B. (2001). Rhesus monkey model for Leishmania major transmitted by Phlebotomus papatasi sandfly bites. Med Vet Entomol.

[46] Grimaldi G. (2008). The utility of rhesus monkey (Macaca mulatta) and other non-human primate models for preclinical testing of Leishmania candidate vaccines. Mem Inst Oswaldo Cruz.

[47] Lainson R., Bray R. (1966). Studies on the immunology and serology of leishmaniasis II. Cross-immunity experiments among different forms of American cutaneous leishmaniasis in monkeys. Trans R Soc Trop Med Hyg.

[48] Dennis V.A., Lujan R. (1986). Leishmania donovani: cellular and humoral immune responses after primary and challenge infections in squirrel monkeys. *Saimiri sciureus.* Exp Parasitol.

[49] Lujan R., Dennis V.A. (1986). Blastogenic responses of peripheral blood leukocytes from owl monkeys experimentally infected with Leishmania braziliensis panamensis. Am J Trop Med Hyg.

[50] Lujan R., Hanson W.L. (1987). Antibody responses, as measured by the enzyme-linked immunosorbent assay (ELISA), in owl monkeys experimentally infected with Leishmania braziliensis panamensis. J Parasitol.

[51] Pung O.J., Kuhn R.E. (1987). Experimental American leishmaniasis in the Brazilian squirrel monkey (Saimiri sciureus): lesions, hematology, cellular, and humoral immune responses. J Med Primatol.

[52] Dube A., Srivastava J.K. (1999). Leishmania donovani: cellular and humoral immune responses in Indian langur monkeys. *Presbytis entellus.* Acta Trop.

[53] Amaral V.F., Pirmez C. (2000). Cell populations in lesions of cutaneous leishmaniasis of Leishmania (L.) amazonensis-infected rhesus macaques, Macaca mulatta. Mem Inst Oswaldo Cruz.

[54] Olobo J.O., Reid G.D.F. (1990). Mitogenic responses of peripheral blood mononuclear cells of vervet monkeys (Cercopithecus aethiops): apparent role of adherent cells. Am J Primatol.

[55] Olobo J.O., Reid G.D.F. (1992). IFN-γ and Delayed-Type Hypersensitivity are Associated with Cutaneous Leishmaniasis in Vervet Monkeys following Secondary Rechallenge with Leishmania major. Scand J Immunol.

[56] Freidag B.L., Mendez S. (2003). Immunological and pathological evaluation of rhesus macaques infected with Leishmania major. Exp Parasitol.

[57] Gicheru M.M., Olobo J.O. (2001). Vervet monkeys vaccinated with killed Leishmania major parasites and interleukin-12 develop a type 1 immune response but are not protected against challenge infection. Infect Immun.

[58] Kenney R.T., Sacks D.L. (1999). Protective immunity using recombinant human IL-12 and alum as adjuvants in a primate model of cutaneous leishmaniasis. J Immunol.

[59] De-Campos S., Souza-Lemos C. (2010). Systemic and compartmentalised immune responses in a Leishmania braziliensis-macaque model of self-healing cutaneous leishmaniasis. Vet Immunol Immunop.

[60] Lemos C.S., Campos S.N.D. (2011). In situ characterization of the granulomatous immune response with time in nonhealing lesional skin of Leishmania braziliensis-infected rhesus macaques (Macaca mulatta). Vet Immunol Immunop.

[61] Campos-Neto A., Porrozzi R. (2001). Protection against cutaneous leishmaniasis induced by recombinant antigens in murine and nonhuman primate models of the human disease. Infect Immun.

[62] Oliveira F., Rowton E. (2015). A sand fly salivary protein vaccine shows efficacy against vector-transmitted cutaneous leishmaniasis in nonhuman primates. Sci Trans Med.

[63] Thacker S.G., McWilliams I.L. (2020). CpG ODN D35 improves the response to abbreviated low-dose pentavalent antimonial treatment in non-human primate model of cutaneous leishmaniasis. PLoS Negl Trop Dis.

[64] Amorim C.F., Novais F.O. (2019). Variable gene expression and parasite load predict treatment outcome in cutaneous leishmaniasis. Sci Trans Med.

[65] Rodrigues V., André S. (2019). Transcriptional analysis of human skin lesions identifies tryptophan-2, 3-deoxygenase as a restriction factor for cutaneous Leishmania. Front Cell Infect Microbiol.

[66] Grimaldi Junior G., Teva A. (2014). Clinical and Parasitological Protection in a Leishmania infantum-Macaque Model Vaccinated with Adenovirus and the Recombinant A2 Antigen. PLoS Negl Trop Dis.

[67] Rodrigues V., Laforge M. (2014). Abortive T follicular helper development is associated with a defective humoral response in Leishmania infantum-infected macaques. PloS Pathog.

[68] Rodrigues V., Cordeiro-da-Silva A. (2012). Modulation of mammalian apoptotic pathways by intracellular protozoan parasites. Cell Microbiol.

[69] Rodrigues V., Cordeiro-da-Silva A. (2014). Impairment of T cell function in parasitic infections. PLoS Negl Trop Dis.

[70] Rodrigues V., Cordeiro-da-Silva A. (2016). Regulation of immunity during visceral Leishmania infection. Parasites & vectors.

[71] Ghalib H.W., Piuvezam M.R. (1993). Interleukin 10 production correlates with pathology in human Leishmania donovani infections. J Clin Invest.

[72] Karp C.L., El-Safi S.H. (1993). In vivo cytokine profiles in patients with kala-azar. Marked elevation of both interleukin-10 and interferon-gamma. J Clin Invest.

[73] Anderson C.F., Oukka M. (2007). CD4+ CD25− Foxp3− Th1 cells are the source of IL-10–mediated immune suppression in chronic cutaneous leishmaniasis. J Exp Med.

[74] Stäger S., Maroof A. (2006). Distinct roles for IL-6 and IL-12p40 in mediating protection against Leishmania donovani and the expansion of IL-10+ CD4+ T cells. Eur J Immunol.

[75] Nylén S., Maurya R. (2007). Splenic accumulation of IL-10 mRNA in T cells distinct from CD4+ CD25+ (Foxp3) regulatory T cells in human visceral leishmaniasis. J Exp Med.

[76] Mesquita I., Ferreira C. (2018). The impact of IL-10 dynamic modulation on host immune response against visceral leishmaniasis. Cytokine.

[77] Resende M., Moreira D. (2013). Leishmania-infected MHC class IIhigh dendritic cells polarize CD4+ T cells toward a nonprotective T-bet+ IFN-γ+ IL-10+ phenotype. J Immunol.

[78] Kim C.H., Rott L.S. (2001). Subspecialization of CXCR5+ T cells: B helper activity is focused in a germinal center–localized subset of CXCR5+ T cells. J Exp Med.

[79] Johnston R.J., Poholek A.C. (2009). Bcl6 and Blimp-1 are reciprocal and antagonistic regulators of T follicular helper cell differentiation. Science.

[80] Nurieva R.I., Chung Y. (2009). Bcl6 mediates the development of T follicular helper cells. Science.

[81] Moukambi F., Rabezanahary H. (2015). Early loss of splenic Tfh cells in SIV-infected rhesus macaques. PloS Pathog.

[82] Moukambi F., Rodrigues V. (2017). CD4 T follicular helper cells and HIV infection: friends or enemies?. Front Immunol.

[83] Linterman M.A., Beaton L. (2010). IL-21 acts directly on B cells to regulate Bcl-6 expression and germinal center responses. J Exp Med.

[84] Zotos D., Coquet J.M. (2010). IL-21 regulates germinal center B cell differentiation and proliferation through a B cell–intrinsic mechanism. J Exp Med.

[85] Polley R., Stager S. (2006). Adoptive immunotherapy against experimental visceral leishmaniasis with CD8+ T cells requires the presence of cognate antigen. Infect Immun.

[86] Murray H.W. (2005). Prevention of relapse after chemotherapy in a chronic intracellular infection: mechanisms in experimental visceral leishmaniasis. J Immunol.

[87] Stäger S., Alexander J. (2003). Both interleukin-4 (IL-4) and IL-4 receptor alpha signaling contribute to the development of hepatic granulomas with optimal antileishmanial activity. Infect Immun.

[88] Laforge M., Silvestre R. (2018). The anti-caspase inhibitor Q-VD-OPH prevents AIDS disease progression in SIV-infected rhesus macaques. J Clin Invest.

[89] Ramesh V., Singh R. (2015). Decline in Clinical Efficacy of Oral Miltefosine in Treatment of Post Kala-azar Dermal Leishmaniasis (PKDL) in India. PLoS Negl Trop Dis.

[90] Rijal S., Ostyn B. (2013). Increasing failure of miltefosine in the treatment of Kala-azar in Nepal and the potential role of parasite drug resistance, reinfection, or noncompliance. Clin Infect Dis.

[91] Abongomera C., Diro E. (2017). The Risk and Predictors of Visceral Leishmaniasis Relapse in Human Immunodeficiency Virus-Coinfected Patients in Ethiopia: A Retrospective Cohort Study. Clin Infect Dis.

[92] van Griensven J., Diro E. (2014). A screen-and-treat strategy targeting visceral leishmaniasis in HIV-infected individuals in endemic East African countries: the way forward?. PLoS Negl Trop Dis.

[93] Diro E., Ritmeijer K. (2018). Long-term Clinical Outcomes in Visceral Leishmaniasis/Human Immunodeficiency Virus-Coinfected Patients During and After Pentamidine Secondary Prophylaxis in Ethiopia: A Single-Arm Clinical Trial. Clin Infect Dis.

[94] Dorlo T.P., Balasegaram M. (2012). Miltefosine: a review of its pharmacology and therapeutic efficacy in the treatment of leishmaniasis. J Antimicrob Chemother.

[95] Rai K., Cuypers B. (2013). Relapse after treatment with miltefosine for visceral leishmaniasis is associated with increased infectivity of the infecting Leishmania donovani strain. mBio.

[96] Mishra J., Singh S. (2013). Miltefosine resistance in Leishmania donovani involves suppression of oxidative stress-induced programmed cell death. Exp Parasitol.

